# Inverse association between remnant cholesterol and risks of atrial fibrillation among patients with type 2 diabetes

**DOI:** 10.3389/fendo.2025.1461613

**Published:** 2025-05-29

**Authors:** Zeyu Jiang, Kun Zhao, Mengqi Sun, Jian Sun, Shuhan Pan

**Affiliations:** ^1^ Department of Emergency Medicine, The Affiliated Hospital of Qingdao University, Qingdao, China; ^2^ Department of Cardiovascular Medicine, The Affiliated Hospital of Qingdao University, College of Medicine, Qingdao University, Qingdao, China; ^3^ Department of Cardiology Medicine, Qingdao Central Hospital, Qingdao, China; ^4^ Department of Pathology, The Affiliated Hospital of Qingdao University, Qingdao, China; ^5^ Department of Neurosurgical Intensive Care Unit, The Affiliated Hospital of Qingdao University, Qingdao, China

**Keywords:** atrial fibrillation, type 2 diabetes mellitus, remnant cholesterol, cardiovascular disease, hyperlipidemia

## Abstract

**Objective:**

We aimed to investigate the association between remnant cholesterol (RC) levels and the risk of atrial fibrillation (AF) among patients with type 2 diabetes, by leveraging data from a large cohort of diabetic patients.

**Methods:**

We included patients with T2DM who received routine care at the affiliated hospital of Qingdao University between January 2014 and December 2023. A total of 9930 patients remained eligible for the final analysis. The primary outcome of this study was the incidence of atrial fibrillation.

**Results:**

During a mean follow up of 6.12 ± 2.75 years, a total of 1,028 AF events occurred. Cox proportional hazards regression was performed to obtain the hazard ratios (HRs) for the incidence of atrial fibrillation by different RC levels at baseline. Compared to Q1, the age- and sex-adjusted HRs for coronary artery disease were 0.89 (95% CI: 0.80-1.28) in Q2, 0.88 (95% CI: 0.80-0.98) in Q3, and 0.76 (95% CI: 0.67-0.84) in Q4. As a continuous variable, RC had an HR of 0.77 (95% CI: 0.64-0.93). After adjusting for age, sex, systolic blood pressure, triglycerides, HbA1c, smoking status, eGFR, and the use of antiplatelet or anticoagulant, lipid-lowering, antihypertensive, and glucose-lowering medications, the multivariable-adjusted HRs were 0.92 (95% CI: 0.82-1.32) in Q2, 0.97 (95% CI: 0.88-1.08) in Q3, and 0.88 (95% CI: 0.79-0.98) in Q4. As a continuous variable, RC had an HR of 0.83 (95% CI: 0.66-0.99).

**Conclusions:**

We demonstrated an inverse association between RC and incident risk of AF using data from real world retrospectively. Future prospective studies with the best option of an interventional trial are needed to further validate our findings.

## 1 Introduction

Atrial fibrillation (AF) is the most common sustained cardiac arrhythmia encountered in clinical practice, significantly contributing to morbidity and mortality worldwide (1). Patients with atrial fibrillation face a fivefold increased risk of stroke and a threefold higher risk of heart failure, with considerable implications for healthcare systems globally (2–5). Atrial fibrillation has diverse risk factors including age, sex, obesity, smoking, type 2 diabetes mellitus (T2DM), hypertension, dyslipidemia, and heart failure. Among these, T2DM has emerged as a significant contributor. The coexistence of T2DM and atrial fibrillation not only amplifies cardiovascular risk but also complicates the management and prognosis of these patients (6–8).

In recent years, attention has turned to the role of lipid abnormalities in the pathogenesis of atrial fibrillation, with remnant cholesterol (RC) being a lipid fraction of particular interest (9, 10). Remnant cholesterol, comprising triglyceride-rich lipoprotein remnants, has been implicated in atherosclerosis and cardiovascular disease independent of traditional lipid parameters such as low-density lipoprotein cholesterol (LDL-C) and high-density lipoprotein cholesterol (HDL-C) (11–13). Despite mounting evidence linking remnant cholesterol to cardiovascular risk, its association with atrial fibrillation, especially in the context of T2DM, remains underexplored especially among Asian population.

T2DM is characterized by a complex interplay of metabolic disturbances, including dyslipidemia, insulin resistance, and chronic inflammation, all of which may contribute to the development of atrial fibrillation. Understanding the specific role of remnant cholesterol in this context is crucial for developing targeted interventions and improving cardiovascular outcomes for patients with T2DM. Therefore, this study aimed to investigate the association between remnant cholesterol levels and the risk of atrial fibrillation among patients with type 2 diabetes, by leveraging data from a large cohort of diabetic patients.

## 2 Methods

### 2.1 Study design and population

The dataset included patients with T2DM who received routine care at the affiliated hospital of Qingdao University between January 2014 and December 2023. We extracted anthropometric, demographic, laboratory, diagnostic, and prescription data from the electronic medical records (EMRs) of these patients. The study design and protocol received written approval from the Institutional Review Boards of the affiliated hospital of Qingdao University (QYFY WZLL 28911). Individual informed consent was not required as we utilized anonymized data sourced from EMRs.

**Figure 1:**
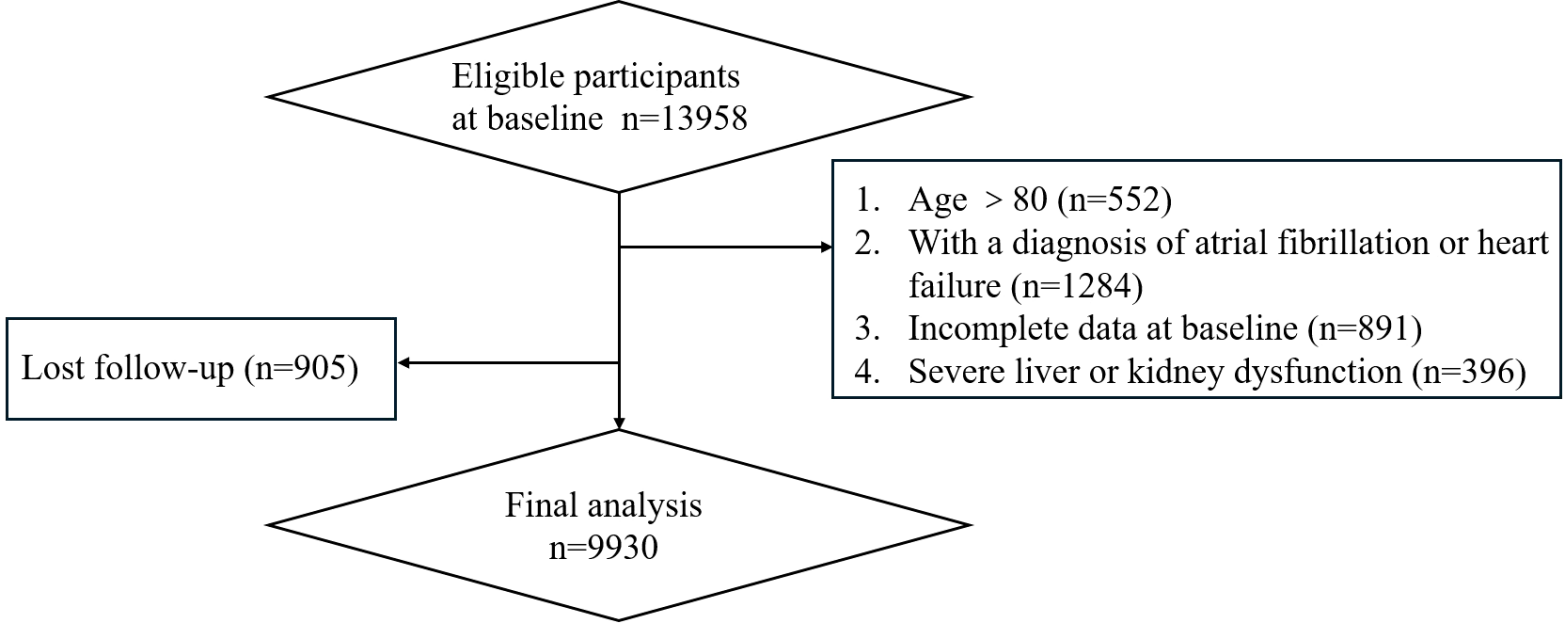
Study flow chart.

The definition of T2DM in this study was based on the SUPREME-DM criteria (14), which include the following: (a) at least one International Classification of Disease, Tenth Revision, Clinical Modification (ICD-10-CM) for T2DM related to inpatient stays; (b) two or more ICD codes associated with outpatient visits on separate days within a two-year period; and (c) a combination of two or more of the following variables related to outpatient visits on different days within two years: ICD codes, fasting glucose level ≥7.0 mmol/L, 2-hour glucose level ≥11.1 mmol/L, random glucose level ≥11.1 mmol/L, HbA1c ≥ 6.5%, and prescription of an glucose-lowering medication. A total of 13,958 patients with T2DM were identified. Patients were further excluded if they were older than 80 years, had a diagnosis of atrial fibrillation or heart failure at baseline, had incomplete data at baseline, or had severe liver or kidney dysfunction. Additionally, patients who were lost to follow-up were also excluded from the final analysis. After applying these criteria, a total of 9,930 patients remained eligible for the final analysis (**Figure 1**).

### 2.2 Patients’ data and measurements

We compiled an analytic database using the unique Chinese Personal Identification Numbers. The database included patients’ information such as date of birth, sex, age at diabetes diagnosis, smoking status, and medication use, including antihypertensive drugs, glucose-lowering drugs, lipid-lowering drugs, and antiplatelet or anticoagulant drugs. Additionally, patients’ height, weight, blood pressure, plasma glucose, C-peptide, lipid profiles including triglycerides (TG), total cholesterol (TC), low-density lipoprotein (LDL) cholesterol, and high-density lipoprotein (HDL) cholesterol, glycated hemoglobin A1c (HbA1c), serum creatinine, and C-reactive protein levels were measured or collected either using standardized methods during inpatient stays or self-report questionnaires during outpatient visits. Body mass index (BMI) was calculated as weight in kilograms divided by height in meters squared (kg/m²). The estimated glomerular filtration rate (eGFR) was determined using the Chronic Kidney Disease Epidemiology Collaboration (CKD-EPI) equation. Information on diabetic complications and comorbidities was extracted from the database and defined according to the International Classification of Diseases, 10th Revision (ICD-10) codes. Patients with a history of atrial fibrillation or heart failure were also identified by ICD-10 codes.

The primary exposure in this study was the calculated remnant cholesterol. The equation for calculating remnant cholesterol is as follows: RC = TC - LDL-C - HDL-C.

### 2.3 Outcomes

The primary outcome of this study was the incidence of atrial fibrillation. Atrial fibrillation was identified either using the ICD-10-CM codes I48.x or based on electrocardiogram (EKG) findings, characterized by the absence of discrete P waves and the presence of irregularly irregular R-R intervals on a 12-lead EKG or a 24-hour Holter monitoring. The incident atrial fibrillation events included both paroxysmal atrial fibrillation and persistent atrial fibrillation.

### 2.4 Statistical analysis

Data were presented differently based on their characteristics: normally distributed data as mean ± standard deviation, skewed data as median with interquartile ranges, and categorical variables as frequencies and percentages. For intergroup comparisons, Student’s t-tests were utilized for normally distributed data, Wilcoxon rank-sum tests for skewed data, and χ2 tests for categorical variables. Fisher’s exact test for categorical variables were further used to verify the results of χ2 tests. Hazard ratios (HRs) for the incidence of atrial fibrillation were estimated using Cox proportional hazards regression, considering RC either as a categorical variable or a continuous variable. RC levels and other categorical variables were treated as dummy variables in the models, with linearity significance tested by assigning ordinal numeric values to each dummy variable. The proportional hazards assumption for the Cox model was evaluated using graphical methods and models incorporating time-by-covariate interactions, and overall, these assumptions were found to be appropriate. Initially, all analyses were performed with adjustments for age and sex, followed by additional adjustments for factors including age, sex, systolic blood pressure, TG, HbA1c, smoking status, eGFR, antiplatelet or anticoagulant, lipid-lowering medications, antihypertensive medications, glucose-lowering medications. Kaplan–Meier analysis was employed to generate survival curves, and event-free survival rates among patients with varying RC levels were compared using the log-rank test. Stratified analyses were conducted based on various patient characteristics, including age, sex, BMI, HbA1c levels, estimated glomerular filtration rate (eGFR), smoking status, and baseline medication use. A P-value of less than 0.05 was considered statistically significant. All statistical analyses were executed using R software version 4.4.1 (R Foundation for Statistical Computing, Vienna, Austria).

## 3 Results

### 3.1 Baseline characteristics

The baseline characteristics of participants according to different RC levels are summarized in **Table 1**. Participants were divided into four quartiles based on RC levels: Q1 (≤0.47 mmol/L), Q2 (0.48–0.64 mmol/L), Q3 (0.65–0.88 mmol/L), and Q4 (>0.88 mmol/L). The mean age was similar across Q1 to Q3, ranging from 66.1 to 66.4 years, but was slightly younger in Q4 at 63.4 years (between-group P<0.05). The proportion of males increased with higher RC levels, from 42.8% in Q1 to 48.8% in Q4 (P for trend < 0.05). Both systolic and diastolic blood pressures were consistent across all quartiles, with systolic around 133 mmHg and diastolic around 76–77 mmHg. BMI increased with higher RC levels, from 23.2 ± 2.41 kg/m² in Q1 to 25.5 ± 4.10 kg/m² in Q4 (P for trend < 0.05). TC, LDL-C, and TG all increased with higher RC levels, whereas HDL-C decreased (all P for trend < 0.05). The eGFR showed a slight decrease from Q1 (94.0 ± 24.9 mL/min/1.73 m²) to Q4 (91.2 ± 24.8 mL/min/1.73 m²) (P for trend < 0.05). HbA1c levels increased from 7.1 ± 1.5% in Q1 to 7.7 ± 1.7% in Q4 (P for trend < 0.05). The percentage of current smokers varied slightly among the quartiles, with the lowest in Q2 (7.3%) and the highest in Q1 (10.4%). The use of antihypertensive medications increased from 71.4% in Q1 to 77.0% in Q4, lipid-lowering medications from 52.9% in Q1 to 66.8% in Q4, and glucose-lowering medications from 67.5% in Q1 to 82.6% in Q4 (all P for trend < 0.05). The use of antiplatelet or anticoagulant medications remained relatively stable across the quartiles.

**Table 1:** Baseline characteristics of participants according to different RC levels.

Characteristics	Remnant cholesterol (mmol/L)			
	Q1 ≤0.47	Q2 0.48~0.64	Q3 0.65-0.88	Q4 >0.88
Participants (n)	2330	2335	2327	2328
Age (years)	66.4 ± 12.2	66.3 ± 11.7	66.1 ± 11.6	63.4 ± 11.6
Male (%)	997 (42.8%)	1036 (44.4%)	1009 (43.4%)	1136 (48.8%)
Blood pressure (mmHg)				
Systolic	123 ± 11	131 ± 13	126 ± 14	129 ± 10
Diastolic	72 ± 7	76 ± 5	73 ± 9	71 ± 8
Body mass index (kg/m^2^)	23.2 ± 2.41	24.0 ± 3.76	24.6 ± 3.19	25.5 ± 4.10
Total cholesterol (mmol/L)	4.14 ± 0.86	4.36 ± 0.85	4.57 ± 0.91	4.91 ± 1.03
Low-density lipoprotein cholesterol (mmol/L)	2.43 ± 0.69	2.62 ± 0.75	2.71 ± 0.81	2.69 ± 0.92
High-density lipoprotein cholesterol (mmol/L)	1.34 ± 0.37	1.19 ± 0.29	1.12 ± 0.26	1.01 ± 0.23
Triglycerides (mmol/L)	0.85 ± 0.21	1.23 ± 0.17	1.66 ± 0.24	1.78 ± 0.56
Estimated GFR (mL/min/1.73 m^2^)	94.0 ± 24.9	92.5 ± 27.1	91.4 ± 25.1	91.2 ± 24.8
HbA1c (%)	7.1 ± 1.5	7.3 ± 1.5	7.4 ± 1.6	7.7 ± 1.7
Current smoking (%)	243 (10.4%)	171 (7.3%)	201 (8.6%)	203 (8.7%)
Use of antihypertensive medications (%)	1664 (71.4%)	1837 (78.7%)	1819 (78.2%)	1793 (77.0%)
Use of lipid-lowering medications (%)	1232 (52.9%)	1468(62.9%)	1509(64.8%)	1554 (66.8%)
Use of glucose-lowering medications (%)	1572 (67.5%)	1768 (75.7%)	1849 (79.5%)	1922 (82.6%)
Use of antiplatelet or anticoagulant (%)	383 (16.4%)	369 (15.8%)	392 (16.8%)	345 (14.8%)

### 3.2 Hazard ratios for the incidence of atrial fibrillation

**Figure 2:**
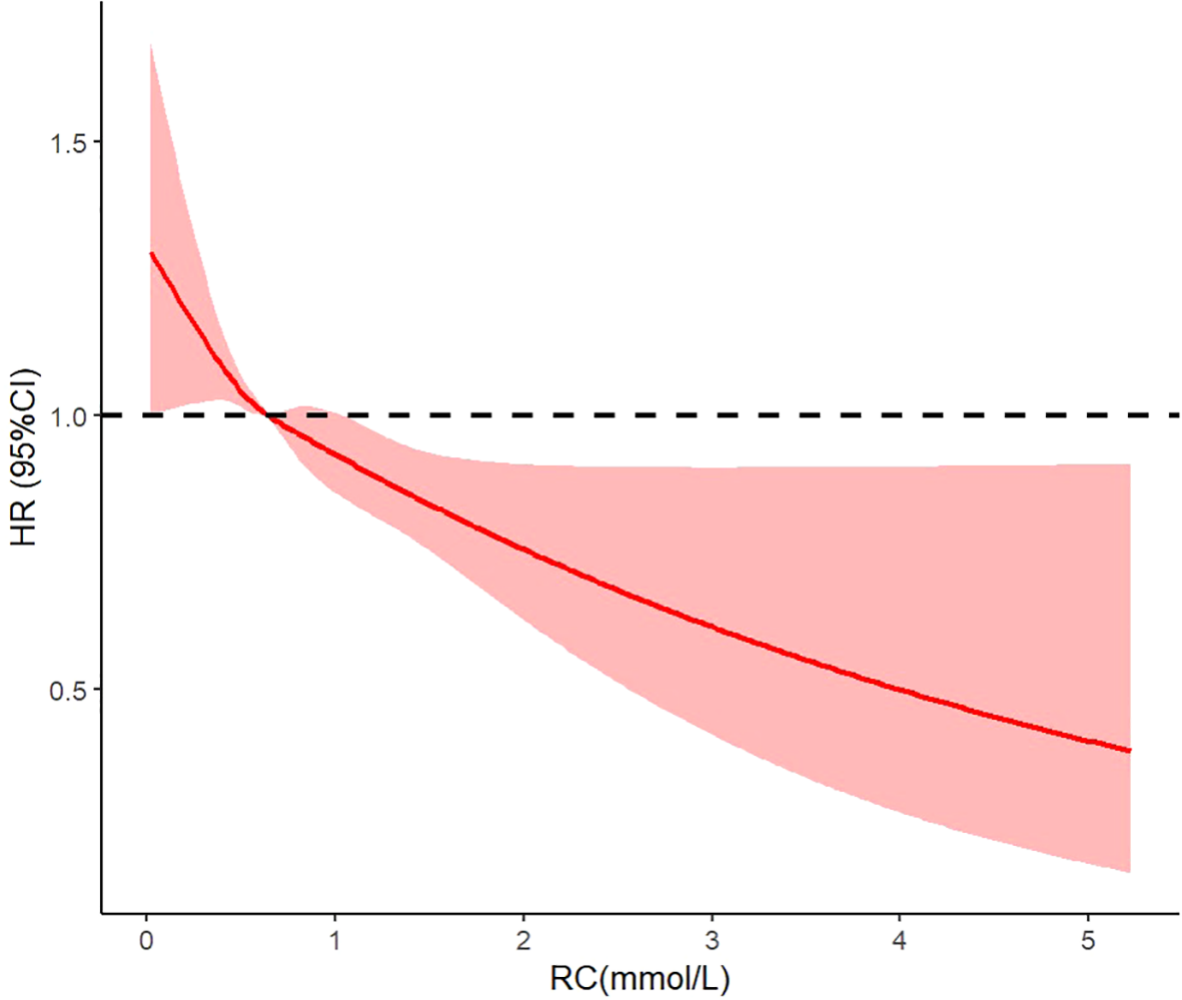
Restricted cubic spline analysis of the association between remnant cholesterol and risks of atrial fibrillation.

During a mean follow up of 6.12 ± 2.75 years, a total of 1,028 AF events occurred. The HRs for the incidence of atrial fibrillation by different RC levels at baseline are presented in **Table 2**. Compared to Q1, the age- and sex-adjusted HRs for coronary artery disease were 0.89 (95% CI: 0.80-1.28) in Q2, 0.88 (95% CI: 0.80-0.98) in Q3, and 0.76 (95% CI: 0.67-0.84) in Q4. Further Kaplan–Meier analysis confirmed the above findings (**Supplementary Figure S1**, Q4 had the lowest incidence of AF). As a continuous variable, RC had an HR of 0.77 (95% CI: 0.64-0.93). After adjusting for age, sex, systolic blood pressure, triglycerides, HbA1c, smoking status, eGFR, and the use of antiplatelet or anticoagulant, lipid-lowering, antihypertensive, and glucose-lowering medications, the multivariable-adjusted HRs were 0.92 (95% CI: 0.82-1.32) in Q2, 0.97 (95% CI: 0.88-1.08) in Q3, and 0.88 (95% CI: 0.79-0.98) in Q4. As a continuous variable, RC had an HR of 0.83 (95% CI: 0.66-0.99) (**Figure 2**).

**Table 2:** Hazard ratios of coronary artery diseases by different levels of remnant cholesterol at baseline.

	Remnant cholesterol (mmol/L)				As a continuous variable
	Q1 ≤0.47	Q2 0.48~0.64	Q3 0.65-0.88	Q4 >0.88	
Coronary artery diseases					
No. of participants	2330	2335	2327	2328	
No. of cases	285	262	260	221	
Age- and sex-adjusted HR	1.00	0.89 (0.80-1.28)	0.88 (0.80-0.98)	0.76 (0.67-0.84) ^a^	0.77 (0.64-0.93)
aHR	1.00	0.92 (0.82-1.32)	0.97 (088-1.08)	0.88 (0.79-0.98) ^a^	0.83 (0.66-0.99)

Multivariable adjusted models included age, sex, systolic blood pressure, TG, HbA1c, smoking status, eGFR, antiplatelet or anticoagulant, lipid-lowering medications, antihypertensive medications, glucose-lowering medications. ^a^ P<0.05.

### 3.3 Subgroup analysis

The association between baseline RC levels and AF was evaluated through subgroup analysis according to various baseline characteristics, as shown in **Supplementary Table S1**. The inverse association between RC and incident AF events were more evident among patients aged 65 years or older, maple patients, patients with HbA1c < 7.0%, patients with eGFR < 60 mL/min/1.73 m^2^, patients without smoking as well as patients with current use of antiplatelet or anticoagulant, lipid-lowering, antihypertensive and glucose-lowering medications. The P for interaction values indicated no significant interactions for age, sex, smoking status, antiplatelet or anticoagulant use, lipid-lowering medication use, antihypertensive medication use, or glucose-lowering medication use. There was a significant interaction for BMI (P for interaction = 0.001) and eGFR (P for interaction = 0.073).

## 4 Discussion

In this study using real-world data from EMRs, we found an inverse association between RC and incident AF events. T2DM patients with higher remnant cholesterol levels tended to have a lower risk of developing atrial fibrillation, especially if they were older, male, had good blood sugar control, poor kidney function, did not smoke, or were using specific medications.

The relationship between cholesterol levels and AF has not been extensively studied, yielding mixed results. Despite the well-documented link between adverse lipid profiles and cardiovascular diseases, a paradoxical association between cholesterol and AF has been observed in several studies. Research has indicated that elevated levels of LDL-C, TC, and HDL-C may be linked to a decreased risk of developing AF. A study investigated the link between RC and AF using data from 392,783 AF-free participants at baseline in the UK Biobank. The authors concluded that low RC levels are independently associated with a higher risk of incident AF.^9^ Another study aimed to clarify these associations in a general population without a history of ASCVD, HF, or AF. Using data from the claims database in Japan involving 1,313,722 individuals, the authors found that remnant cholesterol was positively associated with ASCVD and HF but showed a marginal inverse association with AF (15). A mendelian randomization analysis, however, found no significant associations for low-density lipoprotein cholesterol nor triglycerides associated with the risk of AF. This study provided genetic evidence that lipoprotein a (LPA) may be a causal factor for AF (16). In our study among an Asian population, we found the inverse association between remnant cholesterol and the risk of AF, which was in consistent with the findings from European population. TG in our study had slight relation to AF. After adjusting for confound factors, the relation disappeared between TG and AF, indication that TG alone might had litter prediction for AF in our study.

Although the lipid paradox is associated with the risk of AF, remnant cholesterol, which is the cholesterol content in triglyceride-rich lipoproteins, has several advantages in reflecting CVD risks. Despite the well-established link between LDL-C and CVD risks, patients with low LDL-C levels can still experience residual cardiovascular risks. RC can reflect the cholesterol content in remnants of very low-density lipoproteins (VLDL) and intermediate-density lipoproteins (IDL), providing a direct measure of atherogenic lipoproteins that can contribute to atherosclerosis. Additionally, unlike LDL-C, which is typically measured in the fasting state, RC can reflect the postprandial state, during which atherogenic lipoproteins are more prevalent. Studies have also shown that higher RC levels correlate with markers of insulin resistance, such as the Homeostatic Model Assessment of Insulin Resistance (HOMA-IR), indicating that RC not only reflects but may also contribute to insulin resistance (17).RC is linked to increased levels of inflammatory markers like high-sensitivity C-reactive protein (hs-CRP) and white blood cells (WBCs). Inflammation is a critical component in the pathogenesis of atherosclerosis, where inflammatory cells infiltrate arterial walls, leading to plaque formation and progression. Elevated RC has been shown to promote this inflammatory response, enhancing the development and instability of atherosclerotic plaques, which are precursors to CVD events (17)​.

The clinical significance and underlying mechanisms of this unexpected inverse relationship between lipid levels and AF risk are not yet fully understood. Several potential explanations for this phenomenon could be addressed. Lipid particles, particularly LDL-C, may have a protective structural role in the myocardium (18, 19). Lower levels of these lipids could lead to structural changes in the heart, such as atrial dilation, that predispose to AF. Studies suggest that lipids may influence the structural integrity and function of cardiac cells, potentially affecting the heart’s electrical stability​ (20). Lipids are essential components of cell membranes and precursors for various hormones. Extremely low lipid levels might disrupt normal metabolic and hormonal balances, affecting cardiac function. For example, lipid abnormalities can influence the synthesis of steroid hormones, which play roles in cardiovascular health. Genetic variations that lead to lower lipid levels might also independently increase AF risk (21). Mendelian randomization studies have indicated that certain genetic profiles associated with low LDL-C levels are also linked to a higher risk of AF (22, 23). These genetic factors may influence pathways that contribute to AF risk independently of lipid levels​. Finally, the use of lipid-lowering medications, particularly statins, might also play a role. While statins reduce LDL-C levels and are generally beneficial for cardiovascular health, their effects on AF risk are complex and not fully understood. Some studies suggest that statins may reduce AF risk due to their anti-inflammatory properties, while others indicate a potential for adverse effects in certain populations.

There are several strengths in our study. First, the sample size is large. Secondly, the follow-up duration is long enough to track more outcome events for analysis. We also highlighted the need for clinicians to routinely monitor the cholesterol levels among patients with T2DM and AF and prescribe routine EKG check-ups for these patients. However, several limitations should also be addressed accordingly. First, this study exclusively relied on data sourced from EMRs of patients undergoing routine care at a single center, may limit the representation of the findings to broader populations or diverse healthcare settings. Secondly, we only had robust data for patients with T2DM because these patients received routine monitoring of cholesterol. The results probably could not be applied to other patients without T2DM. We will consider a future study to expand our study population to the general population. Finally, RC levels in this study were determined by equations while several other studies used the same strategy as ours (10, 24, 25). We are also considering a future study which will use direct measurements of RC by enzymatic methods. Last but not least, we did not collect the medicine classed for diabetes, hypertension and dyslipidemia, which may have potential influence on AF.

## 5 Conclusion

In conclusion, we demonstrated an inverse association between RC and incident risk of AF using data from real world retrospectively. Future prospective studies with the best option of an interventional trial are needed to further validate our findings.

## Data Availability

The original contributions presented in the study are included in the article/**Supplementary Material**. Further inquiries can be directed to the corresponding authors.
