# Supplementary material for: Inverse association between remnant cholesterol and risks of atrial fibrillation among patients with type 2 diabetes
Source: Front Endocrinol (Lausanne). 2025 May 29;16:1461613. doi: 10.3389/fendo.2025.1461613 (PMC12158993; doi:10.3389/fendo.2025.1461613)
Supplement: Supplementary file 1 [file Supplementaryfile1.docx]

**Supplementary Table S1**. Subgroup analysis according to different baseline characteristics in association of baseline RC with atrial fibrillation.

|  | Remnant cholesterol (mmol/L) | | | | As a continuous variable | *P* for interaction |
| --- | --- | --- | --- | --- | --- | --- |
|  | Q1  ≤0.47 | Q2  0.48~0.64 | Q3  0.65-0.88 | Q4  ＞0.88 |  |  |
| Age, years old |  |  |  |  |  | 0.176 |
| <65 | 1.00 | 1.15 (0.87-1.43) | 1.05 (0.81-1.34) | 0.97 (0.91-1.05) | 1.06 (0.88-1.28) |  |
| ≥65 | 1.00 | 0.84 (0.75-0.93) | 0.88 (0.79-0.98) | 0.82 (0.73-0.92) | 0.84 (0.74-0.95) |  |
| Sex |  |  |  |  |  | 0.792 |
| Male | 1.00 | 0.87 (0.76-1.00) | 0.84 (0.73-0.96) | 0.62 (0.53-0.71) | 0.61 (0.53-0.70) |  |
| Female | 1.00 | 0.92 (0.79-1.00) | 0.95 (0.83-1.10) | 0.94 (0.81-1.09) | 0.93 (0.81-1.08) |  |
| Body mass index, kg/m^2^ |  |  |  |  |  | 0.001 |
| <25 | 1.00 | 1.06(0.84-1.32) | 0.91 (0.71-1.19) | 0.69 (0.51-0.94) | 0.61 (0.45-0.84) |  |
| 25-29.9 | 1.00 | 0.88 (0.81-0.98) | 0.89 (0.81-0.98) | 0.76 (0.69-0.84) | 0.76(0.68-0.84) |  |
| ≥30 | 1.00 | 0.89 (0.77-1.01) | 0.93 (0.82-1.07) | 0.87 (0.76-0.99) | 0.87 (0.70-0.98) |  |
| HbA1c, % |  |  |  |  |  | 0.964 |
| <7.0 | 1.00 | 0.98 (0.86-1.12) | 0.97 (0.85-1.10) | 0.88 (076-0.96) | 0.80 (0.68-0.94) |  |
| ≥7.0 | 1.00 | 0.87 (0.74-1.02) | 0.96 (0.82-1.12) | 0.90(0.77-1.05) | 1.50 (1.30-1.73) |  |
| Estimated GFR, mL/min/1.73 m^2^ |  |  |  |  |  | 0.073 |
| ≥90 | 1.00 | 1.02 (0.86-1.21) | 1.00 (0.84-1.19) | 0.91 (0.76-1.09) | 0.87 (0.73-1.05) |  |
| 60-89 | 1.00 | 0.98 (0.88-1.) | 1.00 (0.90-1.13) | 0.91 (0.79-0.96) | 1.52 (1.22-1.90) |  |
| <60 | 1.00 | 0.76 (0.61-0.94) | 0.77 (0.62-0.95) | 0.68 (0.55-0.84) | 0.69 (0.56-0.86) |  |
| Current smoking |  |  |  |  |  | 0.189 |
| No | 1.00 | 0.94 (0.85-1.05) | 0.95 (0.86-1.06) | 0.89 (0.80-0.99) | 0.89 (0.80-0.99) |  |
| Yes | 1.00 | 0.67 (0.44-1.00) | 0.95 (0.66-1.38) | 0.69 (0.46-1.02) | 0.67 (0.45-1.00) |  |
| Antiplatelet or anticoagulant |  |  |  |  |  | 0.069 |
| No use | 1.00 | 0.93(0.82-1.06) | 0.94 (0.83-1.07) | 0.87 (0.73.-1.05) | 0.89 (0.78-1.01) |  |
| Use | 1.00 | 092 (0.78-1.10) | 0.98 (0.82-1.17) | 0.88 (0.77-1.00) | 0.83 (0.69-.0.99) |  |
| Lipid-lowering medications |  |  |  |  |  | 0.174 |
| No use | 1.00 | 0.89 (0.75-1.06) | 0.84 (0.70-1.00) | 0.96 (0.80-1.14) | 0.93 (0.77-1.12) |  |
| Use | 1.00 | 0.91 (0.81-1.03) | 0.96 (0.85-1.08) | 0.81(0.71-0.92) | 0.82 (0.72-0.92) |  |
| Antihypertensive medications^‡^ |  |  |  |  |  | 0.359 |
| No use | 1.00 | 0.85 (0.61-1.17) | 0.86 (0.62-1.19) | 1.00 (0.73-1.38) | 0.94 (0.68-0.91) |  |
| Use | 1.00 | 0.88 (0.79-0.98) | 0.90 (0.81-1.01) | 0.81 (0.73-0.91) | 0.83 (0.74-0.92) |  |
| Glucose-lowering medications |  |  |  |  |  | 0.715 |
| No use | 1.00 | 1.06 (0.87-1.30) | 1.01 (0.82-1.25) | 0.84 (0.66-1.06) | 0.88 (0.69-1.12) |  |
| Use | 1.00 | 0.88 (0.78-0.99) | 0.92 (0.82-1.03) | 0.85 (0.76-0.96) | 0.85 (0.76-0.96) |  |

Data are hazard ratios (95% confidence intervals) unless otherwise indicated. Multivariable adjusted models included age, sex, systolic blood pressure, TG, HbA1c, smoking status, eGFR, antiplatelet or anticoagulant, lipid-lowering medications, antihypertensive medications, glucose-lowering medications other than variables for stratification.


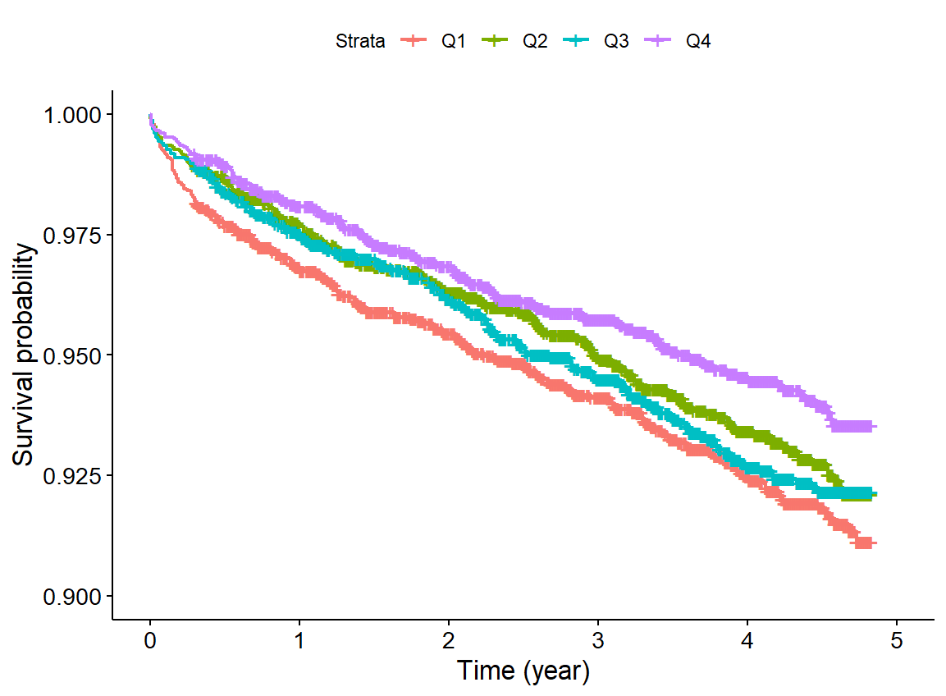


**Supplemental figure S1.** Kaplan-Meier analysis of the association between different quartiles of remnant cholesterol and risks of atrial fibrillation.
